# Interfacial Chemical Effects of Amorphous Zinc Oxide/Graphene

**DOI:** 10.3390/ma14102481

**Published:** 2021-05-11

**Authors:** Zhuo Zhao, Fang Fang, Junsheng Wu, Xinru Tong, Yanwen Zhou, Zhe Lv, Jian Wang, David Sawtell

**Affiliations:** 1 School of Chemical Engineering, University of Science and Technology Liaoning, Anshan 114051, China; zhaozhuo@ustl.edu.cn (Z.Z.); justinwu@yeah.net (J.W.); lz198705222@126.com (Z.L.); 2 Research Institute of Surface Engineering, University of Science and Technology Liaoning, Anshan 114051, China; aiyu912@163.com; 3 Ansteel Iron and Steel Research Institute, Anshan 114009, China; 15566262831@163.com; 4 College of Science, University of Science and Technology Liaoning, Anshan 114051, China; jwang@ustl.edu.cn; 5 Surface Engineering Group, Manchester Metropolitan University, Manchester M15GD, UK

**Keywords:** amorphous, composite film, photoelectric properties, chemical bonds

## Abstract

Research on the preparation and performance of graphene composite materials has become a hotspot due to the excellent electrical and mechanical properties of graphene. Among such composite materials, zinc oxide/graphene (ZnO/graphene) composite films are an active research topic. Therefore, in this study, we used the vacuum thermal evaporation technique at different evaporation voltages to fabricate an amorphous ZnO/graphene composite film on a flexible polyethylene terephthalate (PET). The amorphous ZnO/graphene composite film inherited the great transparency of the graphene within the visible spectrum. Moreover, its electrical properties were better than those of pure ZnO but less than those of graphene, which is not consistent with the original theoretical research (wherein the performance of the composite films was better than that of ZnO film and slightly lower than that of graphene). For example, the bulk free charge carrier concentrations of the composite films (0.13, 1.36, and 0.47 × 10^18^ cm^−3^ corresponding to composite films with thicknesses of 40, 75, and 160 nm) were remarkably lower than that of the bare graphene (964 × 10^18^ cm^−3^) and better than that of the ZnO (0.10 × 10^18^ cm^−3^). The underlying mechanism for the abnormal electrical performance was further demonstrated by X-ray photoelectron spectroscopy (XPS) detection and first-principles calculations. The analysis found that chemical bonds were formed between the oxide (O) of amorphous ZnO and the carbon (C) of graphene and that the transfer of the π electrons was restricted by C=O and C-O-C bonds. Given the above, this study further clarifies the mechanism affecting the photoelectric properties of amorphous composite films.

## 1. Introduction

Zinc oxide (ZnO) films underpin the development of solar cells [1,2], gas sensor applications [3,4,5], displays [6,7,8], and optoelectronic components [9,10,11]; moreover, they can function both as electrodes and as front windows owing to their semiconducting properties and wide band gap. Most of the current studies focus on crystalline ZnO films prepared on a glass substrate. To expand the application field, flexible substrates are replacing glass. Graphene as a flexible substrate, which has a special 2D structure and extraordinary mechanical [12,13,14], optical [15,16,17], and electrical properties [18,19], is a good solution for a wider range of applications, such as light-emitting diodes [20], flexible displays [21], and flexible solar energy [22], due to the fact that it is not limited in terms of shape, light, or flexibility.

The properties of ZnO/graphene composite films are different from those of the individual constituent materials. Experimental studies have been conducted on graphene composites with ZnO nanoparticles or nanorods [23,24,25,26,27,28,29,30]. In these studies, the composite films showed superior photovoltaic [31], photocatalytic [32], photoluminescence [33], antibacterial [34], and sensing [35] properties due to the high transmittance, electron collection enhancement, obstruction of electron–hole pair recombination [29], and good contact between the ZnO and graphene [36]. In general, crystalline ZnO/graphene composites have been regarded as a promising strategy for enhancing the properties of ZnO or graphene.

To understand the underlying mechanism of performance change, some scholars have studied crystalline ZnO/graphene via density functional theory (DFT). The results showed that by changing the layer spacing of ZnO/graphene, the carrier mobility of the system could be modulated [37], and the O-C bond became stronger, with the bond distance decreased from 1.8 Å [38]. Several studies have investigated the connection mechanism of ZnO/graphene and its synthesis, exploring the charge transfer between graphene and ZnO with decreasing distance of the ZnO/graphene layers and the bond formation of ZnO/graphene [26], which is due to synergistic interaction in ZnO/graphene nanocomposites [25].

Most studies have focused on crystalline ZnO/graphene composite films, but amorphous ZnO/graphene composite films remain to be explored. Thus, there is the question as to whether an amorphous ZnO/graphene composite film would retain the high transparency and conductivity of graphene. In this work, through the vacuum thermal evaporation technique, an amorphous ZnO layer was deposited onto graphene/PET to form an ultrathin flexible composite film. 

There are many ways to prepare composite ZnO/graphene films. The cost of magnetron sputtering technology is high, the efficiency of chemical vapor deposition is low, and wastewater treatment by electrochemical plating is difficult and pollutes the environment. However, the vacuum thermal evaporation method has the advantages of low cost, fast evaporation rate, and environmental friendliness. Therefore, the evaporation method is the best choice [39].

After preparation, we carried out relevant detection techniques and the optical and electrical properties of the amorphous ZnO/graphene composite film were characterized to clarify the unexpected results, i.e., the significantly lower electrical properties of the composite films compared with those of the graphene sheet. The interfacial chemical effect of amorphous ZnO/graphene was investigated and analyzed via X-ray photoelectron spectroscopy (XPS) and DFT calculations.

## 2. Experimental Method and Theoretical Calculations

### 2.1. Materials

Graphene/PET composite film was purchased from 2D Carbon Graphene Material Co., Ltd., Changzhou, China (CAS no. of graphene: 1034343-98-0, CAS no. of PET: 25038-59-9). Double-layer graphene was grown via the chemical vapor deposition (CVD) of graphene on a PET substrate with 125 μm thickness. The sheet resistance and visible transparency of the graphene/PET were 157 Ω·sq^−1^ and 90%, respectively. Zinc oxide powder (≥99.99%, 20 mg, CAS no.: 1314-13-2) was purchased from Shanghai Aladdin Biochemical Technology Co., Ltd., Shanghai, China.

### 2.2. Device Fabrication

**Figure 1:**
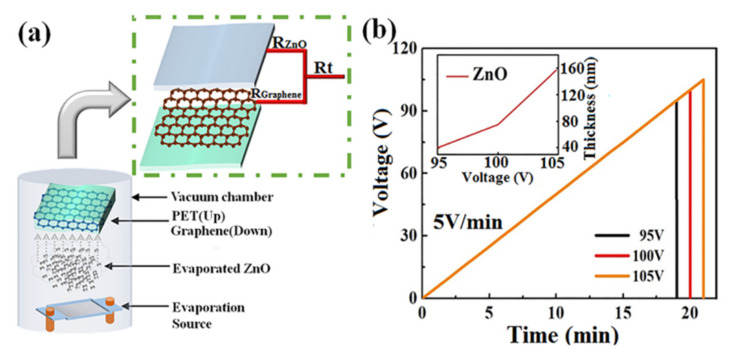
Experimental method and technology: (**a**) vacuum evaporation plating method and a schematic diagram of the parallel resistance of amorphous ZnO/graphene/PET film; (**b**) technological parameter graph of the ZnO deposition process.

The fresh graphene/PET flexible substrate was transferred into the vacuum chamber for ultrathin ZnO layer evaporation at a pressure of 8 × 10^−4^ Pa at room temperature. Zinc oxide powder was placed onto a tungsten (W) boat, which was 160 mm below the substrate. Amorphous ZnO layers with different thicknesses of 40, 75, and 160 nm were thermally evaporated under different evaporation voltages (95, 100, and 105 V), i.e., the voltage applied on the tungsten boat as a heating source, and the rate of voltage increase was 5 V/min. A schematic diagram of the parallel resistance of amorphous ZnO/graphene composite film and the technological parameter graph of the deposition process are shown in Figure 1a,b [40]. The deposition rate of ZnO film increased nonlinearly with the evaporation voltage because the evaporation volume of ZnO powder depended on the temperature of the tungsten boat, which depended on the evaporation voltage—refer to the inset figure in Figure 1b. 

### 2.3. Characterization

#### 2.3.1. Optoelectronic Characterization

The samples were cut into 10 × 10 mm^2^ samples in a square shape. The electrical properties of the composite films were measured using a HALL 8800 Hall-effect measurement device, which was carried out in four-point mode with gold electrodes by DC voltage in a 4800-gauss magnetic field. The transmittance of composite films was measured using a CARY 5000 UV–VIS–NIR spectrometer in the wavelength region of 175–3300 nm (test coverage: 200–800 nm).

#### 2.3.2. Structure and Morphology Characterization

The thicknesses of the ZnO layers were measured using a KLA-Tencor Alpha-Step D-100 profilometer at a speed of 0.03 mm/s and stylus force of 0.03 mg. The roughness of the substrate was measured by atomic force microscopy (AFM CSPM5500), and the roughness was analyzed using Imager software. The samples were scanned in contact mode using a Tap300Al-G probe under ambient atmosphere. The surface morphologies of the film were obtained using a SIGMA high-definition field-emission scanning electron microscope, which was operated at 15 kV. The X-ray diffraction (XRD) patterns of the composite film were recorded using an X’Pert powder X-ray diffractometer with monochromatized Cu Kα radiation. The tube was operated at a scanning speed of 0.05°/s from 20° to 60°. The structures of graphene and ZnO/graphene composite film were characterized by transmission electron microscopy (TEM) at 200 kV. The TEM samples were prepared by scratching the ZnO and graphene layers and placing the minimal debris onto amorphous carbon-coated cooper grids. The element distribution and the interfacial chemical bonds were analyzed via a PHI Quantera SXM scanning photoelectron spectrometer, using an Ar^+^ sputtering gun at 1 kV with an etching rate of 3 nm/min for standard SiO_2_.

### 2.4. First-Principles Calculations

The Vienna ab initio simulation package (VASP) code was used to calculate the electron localization function (ELFCAR) of the interfacial models of amorphous ZnO and graphene. In VASP, the interactions of electrons and ions were described by their projector-augmented wave (PAW) potentials. The generalized gradient approximation parametrized by Perdew–Burke–Ernzerhof was used for the electron exchange–correction function.

In this study, the initial structures of the amorphous ZnO/graphene interface model were investigated. A cluster model of (ZnO)n was selected to represent the amorphous ZnO model. To better match the (ZnO)n clusters and graphene, n was chosen to be 6, so that the model had 6 Zn atoms and 6 O atoms. The cluster model of (ZnO)_6_ was obtained after structural optimization. Similar models have been mentioned in other studies [41,42,43].

The structure of crystalline ZnO (2 × 2 × 1) was obtained from the Materials Project database, with code number 2133; the crystalline ZnO had a P63mc space group symmetry. The structure of graphene (2 × 2) was obtained from the Materials Project database, with the code number 990448; the graphene had a P6/mmm space group symmetry. The valence electron configurations were Zn 3d^10^4s^2^, O 2s^2^p^4^, and C 2s^2^2p^2^.

**Figure 2:**
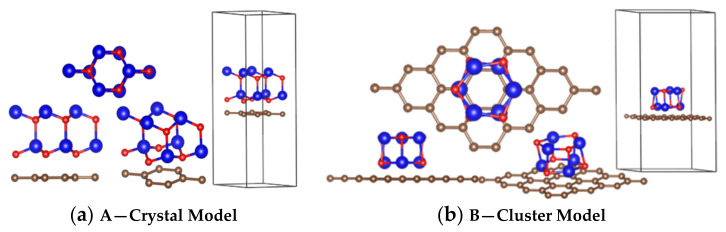
Calculation models: (**a**) crystal ZnO/graphene interface model; (**b**) cluster (amorphous) ZnO/graphene interface model. Color code: Carbon, brown; Zinc, blue; Oxygen, red.

The two interface models were established. Figure 2a shows the crystalline ZnO adjacent to graphene, and Figure 2b shows the amorphous ZnO adjacent to graphene; for both models, the distance between O and C was 1.8 Å.

The cut-off energy in this calculation was 500 eV. The convergence threshold for self-consistent-field iteration was set at 10^−5^ eV, and the width of the Gaussian smearing was 0.05 eV. A Monkhorst-Pack 5 × 5 × 5 k-point mesh was used for the Brillouin zone integration to compute the electron localization function.

## 3. Results and Discussion

### 3.1. Morphology and Phase Structure

**Figure 3:**
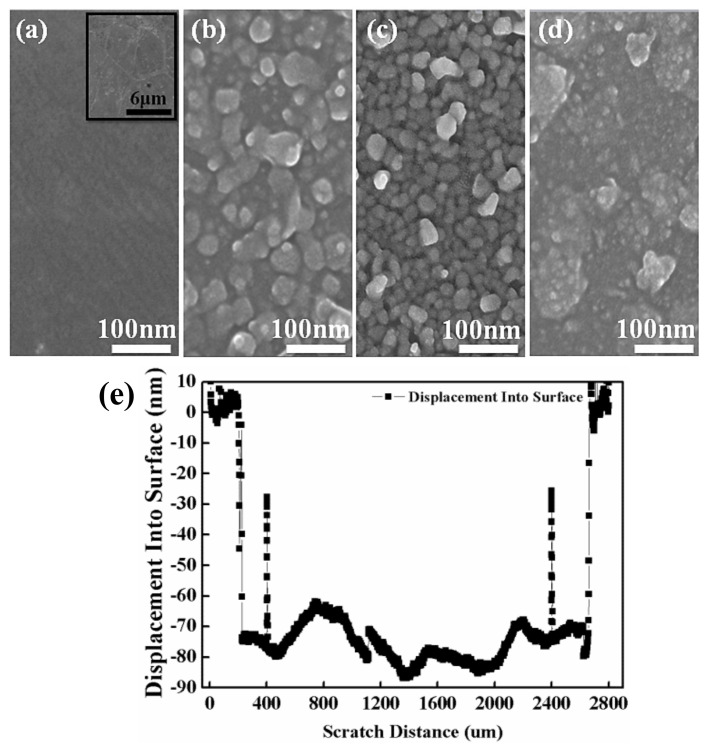
Top-view FESEM images: (**a**) bare graphene/PET and different ZnO films with thickness of (**b**) 40 nm, (**c**) 75 nm, and (**d**) 160 nm which were evaporated onto a graphene/PET substrate at different voltages of 95, 100, and 105 V; (**e**) profilometer scan of ZnO film with 75 nm thickness.

The surface morphological structures of the bare graphene and amorphous ZnO/graphene composite films are displayed in Figure 3. Figure 3a shows the morphology of graphene/PET, and the inset figure in large scale shows the typical morphological structure of bare graphene. At a lower evaporation voltage of 95 V, the amorphous ZnO layer with 40 nm thickness, as measured by the profilometer, showed a loose granular structure (see Figure 3b). A relatively dense granular structure was observed from the ZnO layer with 75 nm thickness, deposited at 100 V (Figure 3c). Finally, the ZnO layer with 160 nm thickness at the highest voltage of 105 V showed a continuous, dense structure with some agglomerates (Figure 3d). The particle sizes, composed of many small particles of the amorphous ZnO films decreased with increasing thickness or evaporation voltage. In other words, the particles were unlikely to cluster as the evaporation voltage increased. 

It is worth mentioning that the range of ZnO particle sizes of the film with 40 nm thickness was from 10 nm to over 50 nm. The roughnesses of the graphene and the films with thickness of 40, 75, and 160 nm were 5, 46, 49, and 77 nm, respectively. This proved that the ZnO film with 40 nm thickness was un-continuous. The ZnO films with thicknesses of 75 nm and 160 nm were dense and continuous.

**Figure 4:**
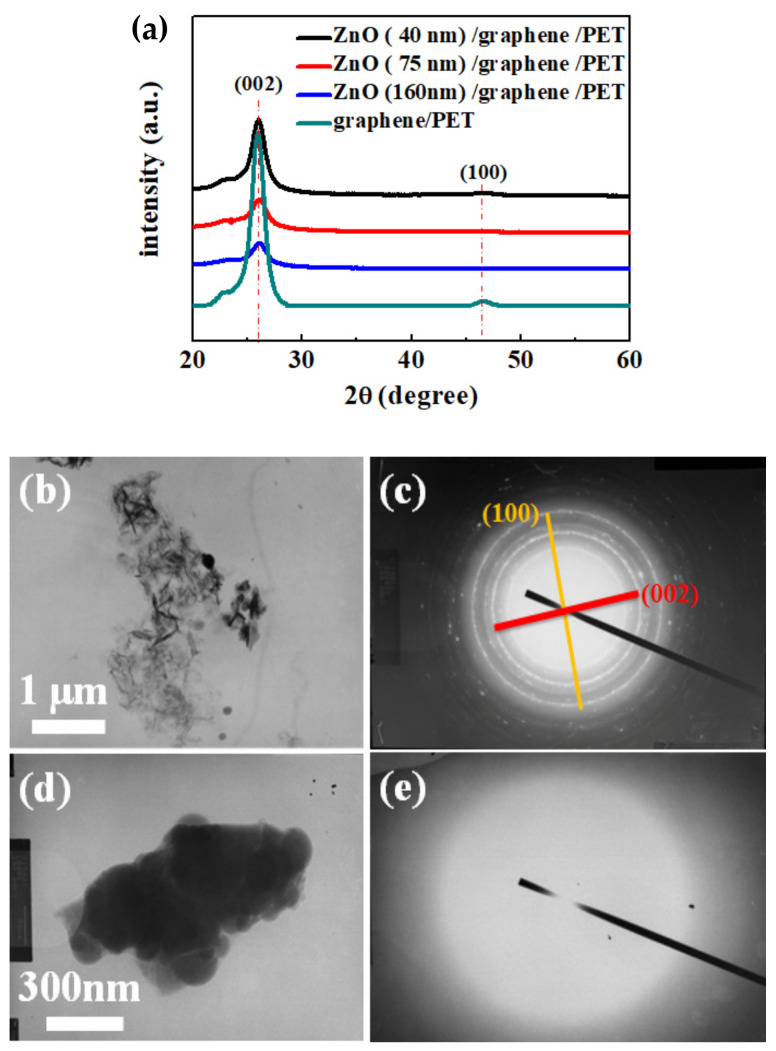
(**a**) X-ray diffraction spectra of graphene/PET and ZnO (40, 75, and 160 nm thickness)/graphene/PET; (**b**,**c**) TEM image and SAED pattern of graphene/PET; (**d**,**e**) TEM image and SAED pattern of ZnO/graphene/PET.

The phase structures of the bare graphene and ZnO/graphene composite film were characterized by XRD, and the results are shown in Figure 4a. Moreover, the TEM morphological images are displayed in Figure 4b,d. The diffraction patterns from selected-area electron diffraction (SAED) of the bare graphene and amorphous ZnO/graphene composite film are given in Figure 4c,e, which correspond to Figure 4b,d, respectively.

From the X-ray diffractometer (XRD) patterns (Figure 4a), the intensity of the bare graphene peak (002) at 26.29° was significant [44]. With the increase in the ZnO layer thickness, the graphene peak intensity decreased. Moreover, the SAED pattern of the TEM result shows the graphene oxide peak (100) at 46.48° (Figure 4c). The graphene oxide peak (100) at 46.48° may be due to weak oxidation of the graphene sample [45]. The SAED pattern showed (002) and (100) polycrystal diffraction circles, which are due to the graphene flakes’ random arrays of graphene debris, caused by the scratching during the TEM sample preparation.

In addition, there is no (002) diffraction peak at 34.40° in Figure 4a and no diffraction circle or patterns of ZnO (002) in Figure 4e; hence, the structure of the ZnO layers deposited on the graphene/PET via thermal evaporation was amorphous.

### 3.2. Optical and Electrical Properties

**Figure 5:**
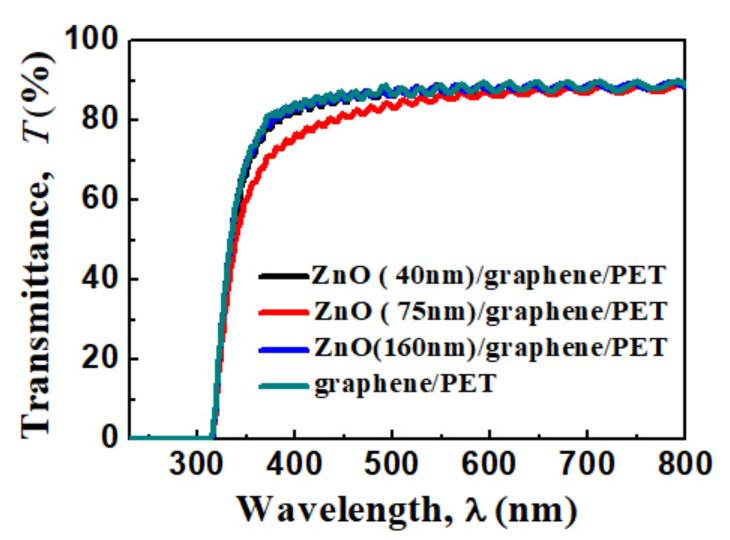
Optical transmittance spectra of graphene/PET and ZnO (40, 75, and 160 nm thickness)/graphene/PET.

The transparent spectra of the graphene/PET and amorphous ZnO/graphene/PET are displayed in Figure 5. The average transparency of the ZnO/graphene/PET composite film was 90% at the 600 nm wavelength range. The slightly lower transparency of the composite film with a 75 nm ZnO layer within the 300–600 nm wavelength might be due to the granular structure (Figure 3c), which resulted in light scattering and absorption functions of the film [46].

Table 1 presents the electrical properties of the graphene/PET, amorphous ZnO/graphene composite film, and pure ZnO. ZnO is likely in the form of molecules or clusters because there is no assistance of an electron or ion beam during the deposition procedure and no ionization process. Therefore, the deficit of oxygen in the ZnO films should be limited, and oxygen vacancies, which would contribute an improvement in electrical properties, are limited. That is the reason why the sheet resistance of the pure ZnO film is as high as 10^5^Ω·sq^−1^, while the carrier concentration and mobility are as low as 10^17^ cm^−3^ and 6-10 cm^2^·v^−1^·s^−1^, respectively.

The continuous and dense ZnO layer makes relatively little contribution to the sheet resistance of the composite film. The parallel resistance of the graphene and ZnO layers was equivalent to that of a single resistor; that is, 1/R_t_ = 1/R_ZnO_ + 1/R_graphene_ [47]. According to the above principle, the predicted composite film sheet resistances should be only slightly less than that of bare graphene. At the same time, some scholars found that the conductivity of crystal ZnO/Graphene composite film increased significantly [48]. However, the composite films’ sheet resistances were almost an order of magnitude greater than the levels for bare graphene. The bulk free charge carrier concentrations of the composite films (10^17–18^ cm^−3^) were remarkably lower than that of bare graphene (10^20^ cm^−3^). To determine the mechanism of the loss of free charge carriers in the ZnO/graphene composite film, we focused on the interfacial chemical effect of amorphous ZnO/graphene. 

### 3.3. The Chemical Bonds at the Amorphous ZnO/Graphene Interface

The above mentioned amorphous ZnO/graphene/PET films exhibited unexpectedly deteriorative electrical properties. The amorphous ZnO/graphene/PET sample with ZnO thickness of 75 nm was selected for XPS analysis, which provided the elemental composition and bonding information.

**Figure 6:**
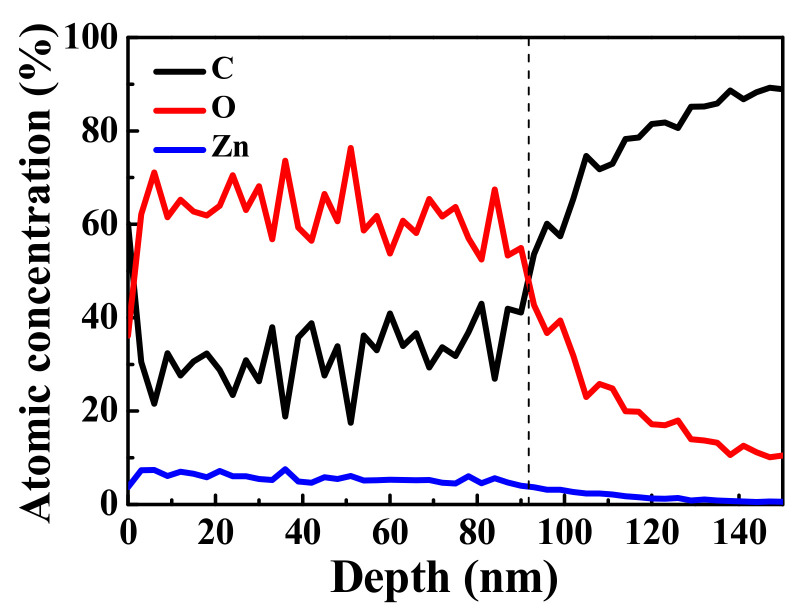
XPS spectra of C, O, and Zn distributions vs. depth (sputtering time) of ZnO/graphene/PET film.

The distributions of C, O, and Zn elements at different depths of the ZnO/graphene/PET composite film are shown in Figure 6. The proportions of C, Zn, and O remained stable up to a depth of about 90 nm. The different thicknesses of the ZnO layer were due to errors in the two measurement techniques. With further sputtering on the composite film up to a depth of 140 nm, only C from the graphene remained. XPS studies on graphene revealed that 1 kV Ar^+^ ion bombardment did not damage the graphene hexahedral structure [49,50,51]; therefore, with the increase in sputtering time, presented as “depth” in Figure 6, O and Zn from ZnO were sputtered away and C from graphene remained. The information of O and Zn at the amorphous ZnO/graphene interface became weak, and that of C was stronger. Thus, it can be concluded that the graphene remained between the amorphous ZnO layer and PET substrate, which was not affected by the evaporation procedure. In other word, the graphene should contribute its conductivity to the composite films.

**Figure 7:**
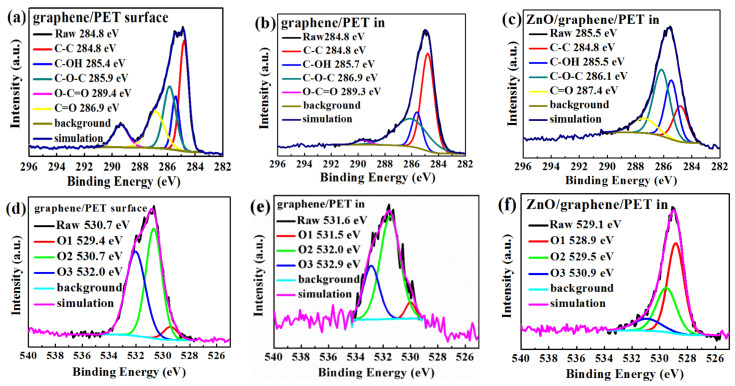
The C1s XPS spectra of the (**a**) graphene surface, (**b**) graphene/PET interface, and (**c**) ZnO/graphene interface; the O1s XPS spectra of the (**d**) graphene surface, (**e**) graphene/PET interface, and (**f**) ZnO/graphene interface.

The XPS C1s and O1s spectra were fitted by the Gaussian–Lorenzian (GL30) functions to reveal the chemical bonding state of the carbon and oxygen (Figure 7a–f). Casa XPS software was applied in this analysis. The analysis result of the graphene surface (Figure 7a) showed the presence of C-C, C-OH, C-O-C, C=O, and O-C=O, resulting from air and graphene [46]. Moreover, C-C, C-OH, and C-O-C occurred at the graphene/PET interface (Figure 7b); in the interface, C=O bonds were absent, and O-C=O and C-O-C bonds became very weak, which means that only graphene and a small quantity of graphene oxide existed [46]. Figure 7c shows the chemical bonds at the interface of amorphous ZnO/graphene/PET, where C-C, C-OH, C-O-C, and C=O bonds existed. The intensity and the integrated area of the C-O-C and C=O peaks at the interface of amorphous ZnO/graphene were much higher and larger than those of the peaks at the graphene/PET interface. The much higher and larger C=O and C-O-C peaks at the interface of amorphous ZnO/graphene suggests that the C from graphene combined with the O from ZnO; therefore, the free π electrons of the graphene were bonded, and the conductivity of the composite films dropped dramatically.

The binding energies of O1 at 530.0–531.6 eV overlapped and were identified as C=O and C-O bonds; the binding energies of O2 and O3 at 532.4–533.3 eV were identified as C-OH [49]. The two O1s positions in the XPS spectra of the graphene surface (Figure 7d) and the graphene/PET interface (Figure 7e) were similar, but the O1 peak at the interface of the amorphous ZnO/graphene/PET (Figure 7f) sharply erupted. This indicates that C=O and C-O bonds were formed at the interface of the amorphous ZnO/graphene again. This evidence further proves that C from graphene combined with O from the amorphous ZnO layer.

One may argue that the oxygens of C=O and C-O came from pollution of the graphene surface because the graphene was used as received, without surface cleaning by the sputtering process. Although it is possible that the oxygen came from surface pollution, the possibility is low, due to the following reasons: First, the graphene was kept in a vacuum bag before use. Second, the fresh graphene was placed in a vacuum chamber, the pressure of which was as low as 10^−4^ Pa; that is, the oxygen not bonded with the C of the graphene was pumped away. Third, the oxygen atoms in the chamber were only provided by ZnO molecules.

When the ZnO powder was evaporated by the vacuum thermal technique, there were ZnO molecules or clusters between the hot tungsten boat and cold graphene/PET substrate. There was not enough energy for ZnO molecules to crystalize, and therefore, the films remained amorphous. The oxide (O) in ZnO molecules was attracted by the carbon (C) in graphene according to the first-principles calculations, which resulted in the formation of C=O and C-O-C bonds.

In general, the formation of C=O and C-O-C bonds at the interface of amorphous ZnO/graphene caused the bonding of the π electrons of the graphene and thereby limited their mobility. In other words, the formation of C=O and C-O-C bonds should be responsible for the decrease in the bulk free charge carriers in the amorphous ZnO/graphene composite film, which is contradictory to earlier studies on crystalline ZnO/graphene composites. Therefore, we believe that the amorphous structure of the ZnO layer is the cause of the formation of the C=O and C-O-C chemical bonds.

### 3.4. Electron Localization Functions

**Figure 8:**
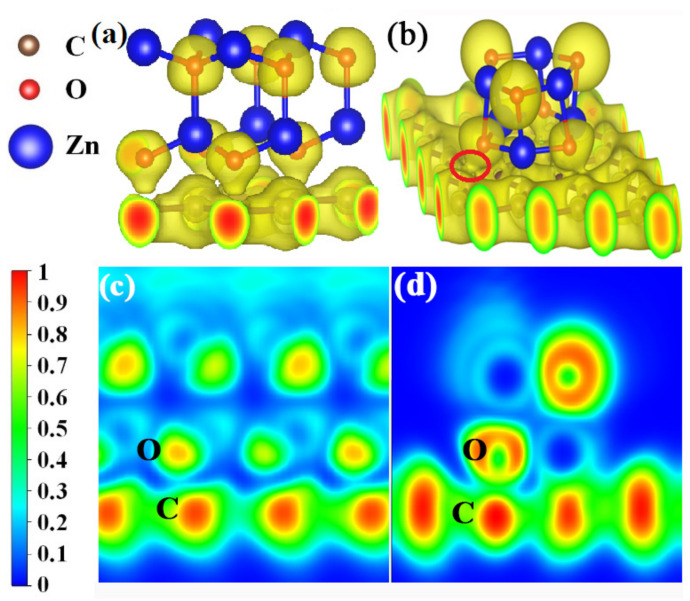
(**a**,**b**) Three-dimensional local electron density distributions with isosurface plots of the ZnO/graphene interface; yellow regions indicate accumulation of electrons. The isosurface plots (with a value of 0.45 eV/bohr^3^) clearly illustrate the electron distribution localized around O atoms. (**c**,**d**) The two-dimensional electron localization function for the ZnO/graphene interface. Blue regions are domains of low electron localization, while red regions are domains of high electron localization. ELF values of 0 and 1 correspond to perfect delocalization and localization, respectively.

The electron localization functions (ELFs) were calculated to further investigate the bonding natures at the crystal (Figure 8a) and amorphous (Figure 8b) ZnO/graphene interfaces. It can be seen from Figure 8a,b that the electron clouds are localized around O atoms (yellow color) and delocalized from Zn atoms, whether in the crystal ZnO model A or the amorphous ZnO model B, which indicates the ionic bonds of ZnO. Also, the covalent C-C bond in graphene can be found in Figure 8, in which the electron localization density of graphene is shown to be fair. The clear distinctions between the two models are as follows: The localized electron clouds C and O are separated between crystal ZnO and graphene, and there is no bond formed between the O of crystalline ZnO and the C of graphene (Figure 8a,c). The localized electron cloud of O from amorphous ZnO joins together with that of C from graphene, which indicates the formation of covalent bonds between the O of amorphous ZnO and the C of graphene (Figure 8b,d). In other words, the crystalline ZnO and graphene do not form chemical bonds, while C-O covalent bonds are formed between the amorphous ZnO and graphene.

## 4. Conclusions

In this study, amorphous ZnO layers on flexible graphene/PET substrates were prepared via vacuum thermal evaporation at room temperature. With the increase in the amorphous ZnO layer thickness, the morphological structure of the layer changed from loose un-continuous granular, to relatively dense granular, to continuous and dense. The transparency of the composite films within the visible spectrum was comparable to that of graphene/PET. Its electrical properties were better than those of the pure ZnO but less than those of graphene. For example, the bulk free charge carrier concentrations of the composite films (0.13, 1.36, and 0.47 × 10^18^ cm^−3^ corresponding to composite films with thickness of 40, 75, and 160 nm) were remarkably lower than that of bare graphene (964 × 10^18^ cm^−3^) and better than that of ZnO (0.10 × 10^18^ cm^−3^). The results from XPS analysis showed the formation of C=O and C-O-C bonds between O from ZnO and C from graphene at the interface of ZnO and graphene. The first-principles calculations revealed that the oxide from amorphous ZnO tended to be attracted by C from graphene to form strong, dense localized electron clouds. The electrical properties of the composite films deteriorated because of the formation of the C=O and C-O-C chemical bonds at the interface of amorphous ZnO/graphene, which restrained the transfer of the π electrons from graphene. This discovery is still helpful for the development of transparent conductive films.

## Figures and Tables

**Table 1:** Electrical properties of the amorphous ZnO/graphene composite film.

Thickness of ZnO Layers, nm	0	40	75	160	Pure ZnO
Sheet resistance, ×10^2^ Ω·sq^−1^	1.57	55.30	12.70	9.06	1000
Carrier concentration, ×10^18^ cm^−3^	964.00	0.13	1.36	0.47	0.10
Carrier mobility, ×10^2^ cm^2^·v^−1^·s^−1^	6.06	20.40	4.81	9.09	0.06–0.10

## Data Availability

The data presented in this study are available on request from the corresponding author.
